# Supplementary figures and images for: Genetic differentiation and phylogeography of Mediterranean-North Eastern Atlantic blue shark (Prionace glauca, L. 1758) using mitochondrial DNA: panmixia or complex stock structure?
Source: PeerJ. 2017 Dec 6;5:e4112. doi: 10.7717/peerj.4112 (PMC5723133; doi:10.7717/peerj.4112)

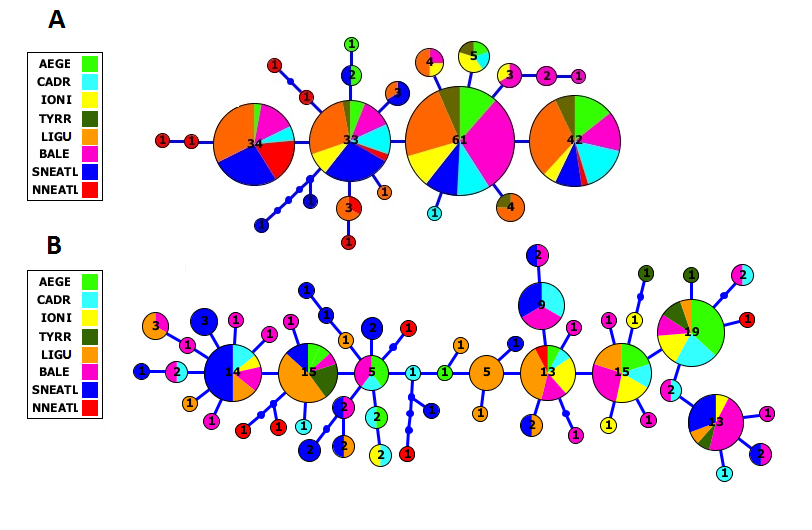

Supplement: Figure S1 [file peerj-05-4112-s001.png]

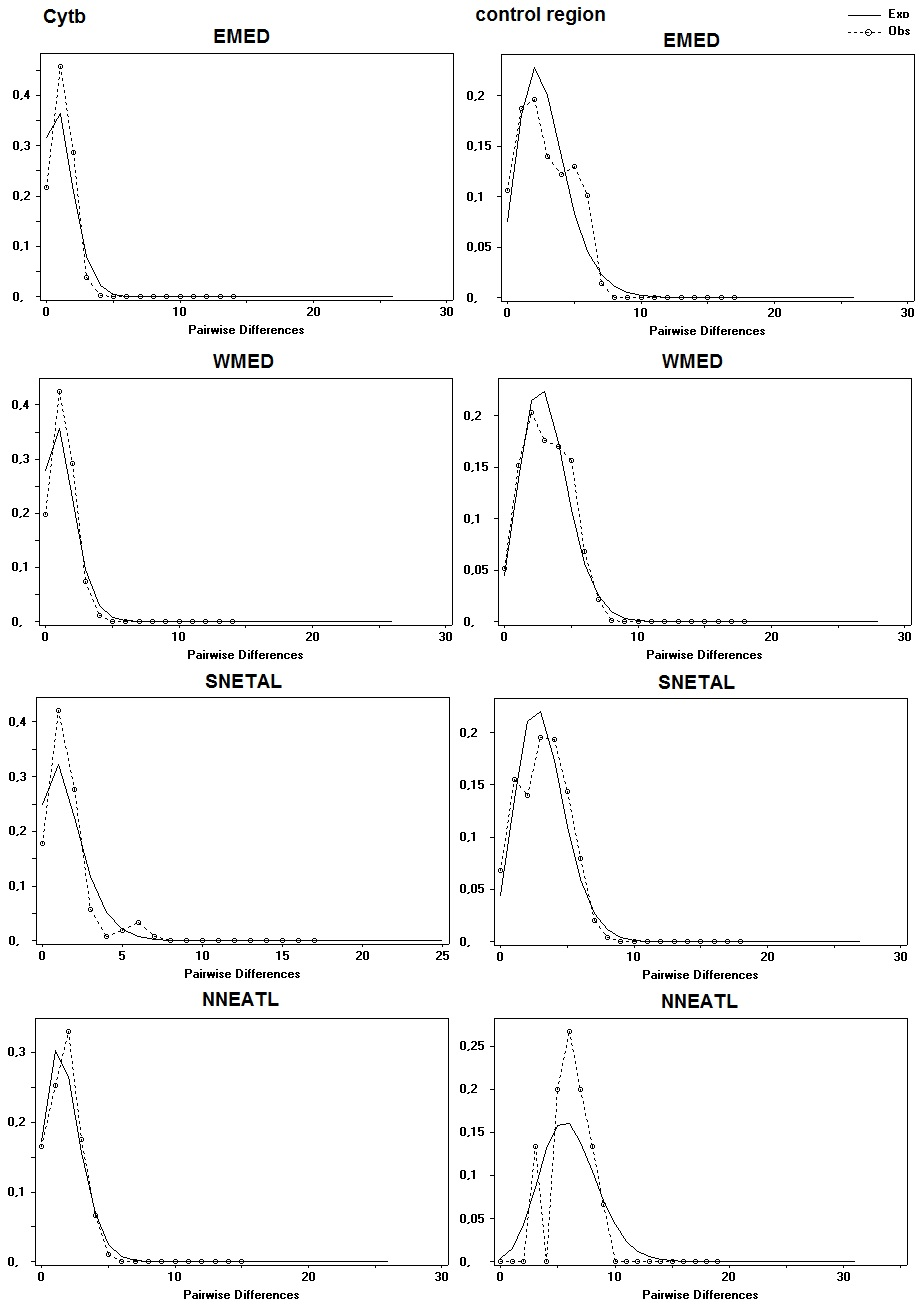

Supplement: Figure S2 [file peerj-05-4112-s002.png]

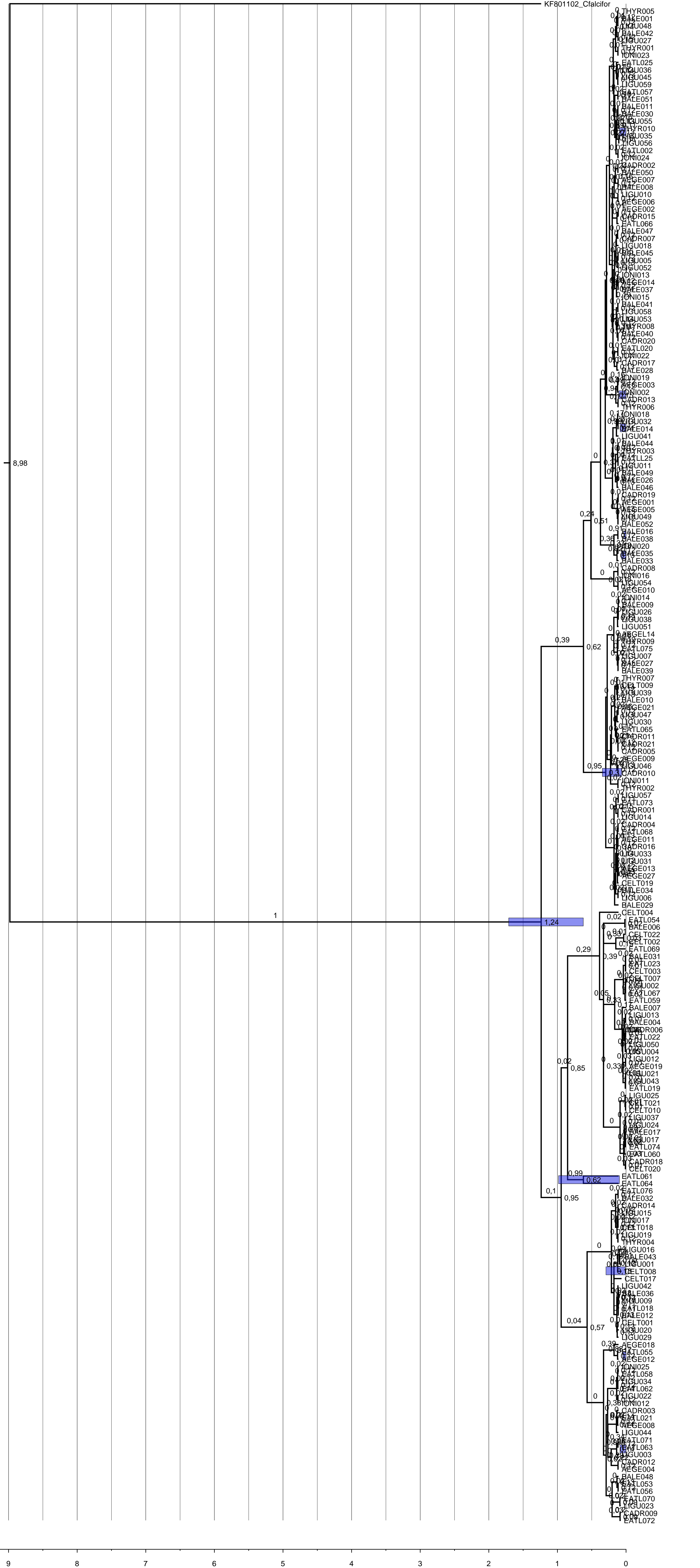

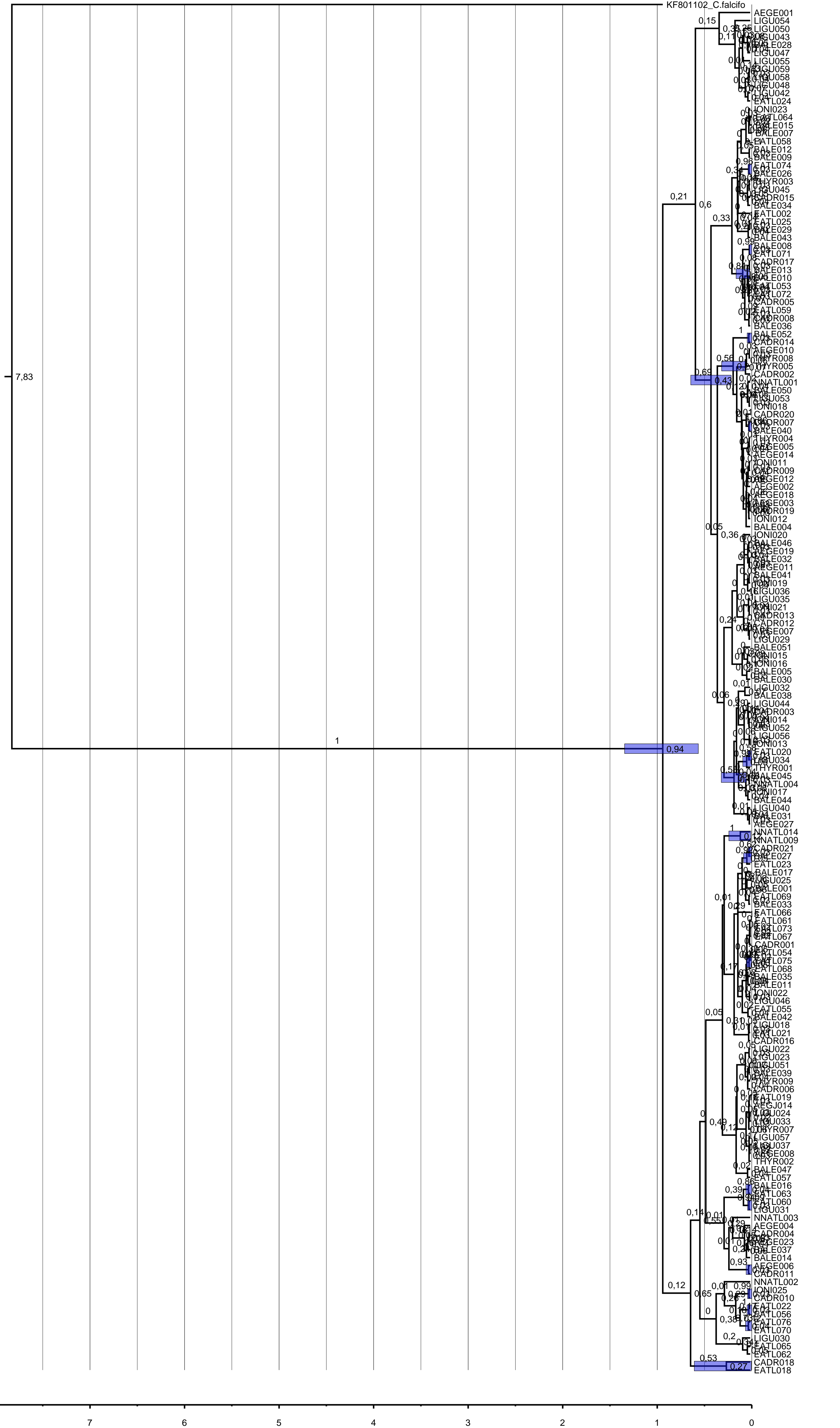

Supplement: Figure S3 — Node ages on the node labels. Posterior probability on the branches. 95% HPD bars showed for nodes with Posterior > 0.5. [file peerj-05-4112-s003.pdf]
