# Supplementary material for: Genetic differentiation and phylogeography of Mediterranean-North Eastern Atlantic blue shark (Prionace glauca, L. 1758) using mitochondrial DNA: panmixia or complex stock structure?
Source: PeerJ. 2017 Dec 6;5:e4112. doi: 10.7717/peerj.4112 (PMC5723133; doi:10.7717/peerj.4112)
Supplement: Table S1 — M, males; F, females; na, unsexed; J, juvenile (TL ≤ 120 cm); Y, young (TL = 120–180 cm); L, large (TL ≥ 180 cm). NATL: North-eastern Atlantic; WMED: Western Mediterranean; EMED: Eastern Mediterranean. [file peerj-05-4112-s004.docx]

|  | NNEATL | SNEATL | BALE | LIGU | TYRR | IONI | AEGE | CADR | NATL | WMED | EMED |
| --- | --- | --- | --- | --- | --- | --- | --- | --- | --- | --- | --- |
| M | 2 | 9 | 32 | 21 | 3 | 6 | 8 | 10 | 11 | 56 | 24 |
| F | 14 | 18 | 10 | 36 | 1 | 4 | 7 | 11 | 32 | 47 | 22 |
| na | 0 | 6 | 0 | 0 | 6 | 5 | 5 | 0 | 6 | 6 | 10 |
| Total | 16 | 33 | 42 | 57 | 10 | 15 | 20 | 21 | 49 | 109 | 56 |
| Total sexed | 16 | 27 | 42 | 57 | 4 | 10 | 15 | 21 | 43 | 103 | 46 |
| %M | 13% | 33% | 76% | 37% | 75% | 60% | 53% | 48% | 26% | 54% | 52% |
| %F | 88% | 67% | 24% | 63% | 25% | 40% | 47% | 52% | 74% | 46% | 48% |
| Sex-ratio | 0.14 | 0.50 | 3.20 | 0.58 | 3.00 | 1.50 | 1.14 | 0.91 | 0.34 | 1.19 | 1.09 |
|  |  |  |  |  |  |  |  |  |  |  |  |
| J | 0 | 8 | 0 | 11 | 9 | 10 | 5 | 20 | 8 | 20 | 35 |
| Y | 2 | 19 | 21 | 33 | 0 | 3 | 3 | 1 | 21 | 54 | 7 |
| L | 9 | 6 | 21 | 13 | 1 | 2 | 12 | 0 | 15 | 35 | 14 |
| Total sized | 11 | 33 | 42 | 57 | 10 | 15 | 20 | 21 | 44 | 109 | 56 |
| %J | 0% | 24% | 0% | 19% | 90% | 67% | 25% | 95% | 18% | 18% | 63% |
| %Y | 18% | 58% | 50% | 58% | 0% | 20% | 15% | 5% | 48% | 50% | 13% |
| %L | 82% | 18% | 50% | 23% | 10% | 13% | 60% | 0% | 34% | 32% | 25% |
