# Supplementary material for: Genetic differentiation and phylogeography of Mediterranean-North Eastern Atlantic blue shark (Prionace glauca, L. 1758) using mitochondrial DNA: panmixia or complex stock structure?
Source: PeerJ. 2017 Dec 6;5:e4112. doi: 10.7717/peerj.4112 (PMC5723133; doi:10.7717/peerj.4112)
Supplement: Table S2 — South North East Atlantic, SNEATL; North North East Atlantic, NNEATL; West-Mediterranean/Balearic, BALE; South Tyrrhenian Sea, TYRR; Calabria Ionian Sea, IONI; Central Adriatic Sea, CADR; Aegean Sea & Eastern Ionian Greece. [file peerj-05-4112-s005.docx]

A

| POP | *N* | *Nh* | *S* | *h* | *stdev h* | *π* | *stdev π* |
| --- | --- | --- | --- | --- | --- | --- | --- |
| NNEATL | 14 | 9 | 6 | 0.835 | 0.101 | 0.00231 | 0.00046 |
| SNEATL | 33 | 8 | 10 | 0.822 | 0.034 | 0.00200 | 0.00038 |
| BALE | 39 | 8 | 5 | 0.749 | 0.059 | 0.00162 | 0.00022 |
| LIGU | 56 | 9 | 5 | 0.828 | 0.022 | 0.00171 | 0.00011 |
| TYRR | 10 | 5 | 4 | 0.800 | 0.100 | 0.00140 | 0.00030 |
| IONI | 15 | 6 | 5 | 0.810 | 0.078 | 0.00145 | 0.00025 |
| AEGE | 19 | 7 | 3 | 0.784 | 0.067 | 0.00158 | 0.00024 |
| CADR | 21 | 6 | 4 | 0.795 | 0.051 | 0.00149 | 0.00017 |
| TOTAL | 207 | 23 | 16 | 0.821 | 0.013 | 0.00184 | 0.00010 |

B

| POP | *N* | *Nh* | *S* | *h* | *stdev h* | *π* | *stdev π* |
| --- | --- | --- | --- | --- | --- | --- | --- |
| NNEATL | 6 | 6 | 15 | 1.000 | 0.096 | 0.00812 | 0.00106 |
| SNEATL | 33 | 17 | 13 | 0.932 | 0.026 | 0.00424 | 0.00038 |
| BALE | 39 | 25 | 16 | 0.962 | 0.017 | 0.00444 | 0.00033 |
| LIGU | 32 | 14 | 9 | 0.907 | 0.029 | 0.00323 | 0.00028 |
| TYRR | 8 | 5 | 8 | 0.857 | 0.108 | 0.00488 | 0.00062 |
| IONI | 14 | 8 | 9 | 0.901 | 0.052 | 0.00319 | 0.00078 |
| AEGE | 17 | 7 | 6 | 0.809 | 0.079 | 0.00309 | 0.00047 |
| CADR | 21 | 14 | 10 | 0.948 | 0.031 | 0.00463 | 0.00037 |
| TOTAL | 170 | 55 | 27 | 0.951 | 0.006 | 0.00453 | 0.00014 |
