# Supplementary material for: Genetic differentiation and phylogeography of Mediterranean-North Eastern Atlantic blue shark (Prionace glauca, L. 1758) using mitochondrial DNA: panmixia or complex stock structure?
Source: PeerJ. 2017 Dec 6;5:e4112. doi: 10.7717/peerj.4112 (PMC5723133; doi:10.7717/peerj.4112)
Supplement: Table S3 [file peerj-05-4112-s006.docx]

|  | Cytb |  |  | |  | | CR | |  |  |
| --- | --- | --- | --- | --- | --- | --- | --- | --- | --- | --- |
|  | % variation | Φ-Statistics | p |  | | % variation | | Φ-Statistics | | p |
| *AMOVA1: Overall*  *(all population samples)* | |  |  |  | |  | |  | |  |
| Among populations | 4.81 |  |  |  | | 11.30 | |  | |  |
| Within populations | 95.19 | ST = 0.0481 | 0.0079 |  | | 88.70 | | ST = 0.11302 | | 0.00000 |
| *AMOVA2: 2 groups:*  *(SNEATL vs WMED+EMED)* | | | |  | |  | |  | |  |
| Among groups | 10.69 | CT = 0.1069 | 0.1403 |  | | 11.87 | | CT = 0.1187 | | 0.14035 |
| Among pops within group | 0.58 | SC = 0.0065 | 0.3115 |  | | 5.72 | | SC = 0.0649 | | 0.00489 |
| Within populations | 88.72 | ST = 0.1128 | 0.0084 |  | | 82.41 | | ST = 0.1759 | | 0.00000 |
| *AMOVA3: 3 groups:*  *(SNEATL vs WMED vs EMED)* | | | | | | | |  | |  |
| Among groups | 5.06 | CT = 0.0506 | 0.2095 |  | | 9.87 | | CT = 0.0987 | | 0.02912 |
| Among pops within group | 0.97 | SC = 0.0103 | 0.2666 | |  | | 3.48 | | SC = 0.0386 | 0.05584 |
| Within populations | 93.97 | ST = 0.0603 | 0.0072 | |  | | 86.65 | | ST = 0.1335 | 0.00000 |
