# Supplementary material for: Genetic differentiation and phylogeography of Mediterranean-North Eastern Atlantic blue shark (Prionace glauca, L. 1758) using mitochondrial DNA: panmixia or complex stock structure?
Source: PeerJ. 2017 Dec 6;5:e4112. doi: 10.7717/peerj.4112 (PMC5723133; doi:10.7717/peerj.4112)
Supplement: Table S4 — *Values that resulted not significant after the Bonferroni correction for multiple tests (a-level of significance after Bonferonni correction: p = 0.0166). [file peerj-05-4112-s007.docx]

|  |  |  | |  | | |
| --- | --- | --- | --- | --- | --- | --- |
| Cytb | SNEATL | | WMED | | EMED |  |
| SNEATL |  | | 0.0025 | | 0.0004 |  |
| WMED | 0.0912 | |  | | 0.4341 |  |
| EMED | 0.1402 | | -0.0019 | |  |  |
|  |  |  | |  | | |
|  |  |  | |  | | |
| CR | SNEATL | | WMED | | EMED |  |
| SNEATL |  | | 0.0004 | | 0.0000 |  |
| WMED | 0.1202 | |  | | 0.0066 |  |
| EMED | 0.2170 | | 0.0605 | |  |  |
